# Practical insights from implementing a national programme for monitoring appropriateness of antimicrobial prescribing in the South-East Asia Region: experience from the CAPTURA project in Bangladesh, Bhutan, and Nepal

**DOI:** 10.1016/j.ijregi.2026.100947

**Published:** 2026-07-10

**Authors:** Ron Cheah, Karin Thursky, Jyoti Acharya, Andréia Regina Augusto dos Santos, Sangita Shrestha, Dorji Tshering, Gitanjali Lamichaney, Mohammad Julhas Sujan, Hridika Talukder Barua, Sonam Zangmo, Sanju Maharjan, Samsad Rabbani Khan, Siddhartha Dhungana, Kumari Kamala Bist, Pem Chuki, Khando Wangchuk, Abul Hasnat, Sooyoung Kwon, Zakir Hossain Habib, Santosh Paudel, Manish Gautam, Freddy Lopez, Pradip Mishra, Nimesh Poudyal, Rodney James

**Affiliations:** 1 The National Centre for Antimicrobial Stewardship, Department of Infectious Diseases, Melbourne Medical School, The Peter Doherty Institute, Melbourne, Australia; 2 RMH Guidance Group, The Royal Melbourne Hospital, Melbourne, Australia; 3 Bir Hospital, National Academy of Medical Sciences, Kathmandu, Bagmati Province, Nepal; 4 International Vaccine Institute, Seoul, Republic of Korea; 5 Phuentsholing General Hospital, National Medical Services, Ministry of Health, Royal Government of Bhutan, Bhutan; 6 Jigme Dorji Wangchuck National Referral Hospital, National AMS and IPC Center, Ministry of Health, Royal Government of Bhutan, Bhutan; 7 Anweshan Private Limited, Health Unit, Lalitpur, Bagmati Province, Nepal; 8 Communicable Disease Control (CDC), Directorate General of Health Services, Ministry of Health and Family Welfare, Dhaka, Bangladesh; 9 Seti Provincial Hospital, Dhangadi, Sudurpaschim Province, Nepal; 10 Institute of Epidemiology, Disease Control and Research (IEDCR), Directorate General of Health Services, Ministry of Health and Family Welfare (MoHFW), Dhaka, Bangladesh

**Keywords:** Antimicrobial stewardship, Low- and middle-income countries, AMS, AMU, AMR, Antimicrobial resistance

## Abstract

**Objectives:**

Surveillance of antimicrobial prescribing quality is a core element of national antimicrobial resistance containment strategies. The National Antimicrobial Prescribing Survey (NAPS) programme was implemented in Bangladesh, Bhutan, and Nepal under the Fleming Fund–supported Capturing Data on Antimicrobial Resistance Patterns and Trends in Use in Regions of Asia project to strengthen national surveillance and stewardship capacity. The programme comprises setting-specific modules using standardized indicators including documentation quality, guideline concordance, and appropriateness.

**Methods:**

Between September 2024 and September 2025, Hospital NAPS point-prevalence audits were conducted in Bangladesh and Nepal, and Surgical NAPS was piloted in Bhutan. Audits were conducted in two phases to enable local interventions between rounds, incorporating stakeholder engagement, structured auditor training, and central quality assurance.

**Results:**

Ten hospitals completed audits encompassing 1299 patients (Hospital NAPS), 236 procedures (Surgical NAPS), and 2308 prescriptions. The programme produced baseline prescribing appropriateness data for the first time to inform stewardship priorities across district to tertiary settings. Key enablers included national engagement and establishment of antimicrobial stewardship committees; barriers included incomplete documentation, infrastructure limitations, and coordination challenges.

**Conclusion:**

The NAPS programme demonstrated feasibility across diverse health system contexts in three South-East Asia Region countries. Sustained leadership, routine practice integration, local ownership, and committed cross-institutional collaboration are critical for long-term stewardship impact.

## Highlights

• The National Antimicrobial Prescribing Survey ‘appropriateness’ metric extends beyond guideline concordance.
• First national ‘appropriateness’ data were generated in Bangladesh and Nepal.
• Antimicrobial use surveillance extended into the surgical prophylaxis setting in Bhutan.
• Barriers were infrastructure and logistical limitations, documentation gaps, and hierarchies.
• Success indicators were national commitment, ownership, and values-aligned collaborations.

## Introduction

Antimicrobial resistance (AMR) is a global health threat that undermines the effective treatment of infections and threatens patient safety [1]. Antimicrobial stewardship (AMS) has emerged as a critical strategy to preserve antimicrobial efficacy [2]. The World Health Organization (WHO) defines AMS as ‘a coherent set of actions which promote the responsible use of antimicrobials’. Surveillance of antimicrobial use (AMU) and antimicrobial consumption (AMC) is a critical component of AMS and national AMR containment strategies. However, many low- and middle-income countries (LMICs) lack standardized mechanisms and resources to conduct AMU surveillance and assess prescribing quality [3]. Therefore, AMC, a simpler metric which quantifies the volume of antimicrobial prescribing, is the most commonly reported surveillance indicator, enabling comparisons across different timeframes, institutions, and countries [4]. However, volume-based metrics alone do not capture whether prescribing is clinically appropriate or concordant with treatment guidelines. An overall decrease in antimicrobial prescribing volume does not reflect responsible, or ‘appropriate’, prescribing or account for patient complexity. By incorporating clinical judgment, patient-specific factors, and evolving local AMR epidemiology, assessments of prescribing appropriateness provide a more nuanced and clinically meaningful assessment of stewardship effectiveness and represent a more relevant indicator for improving patient safety and equity across diverse healthcare settings [5].

There is currently no internationally agreed definition of appropriateness. Some studies report guideline concordance as a single indicator of appropriate antimicrobial therapy [6,7]; however, this is not a direct surrogate for optimal AMU [5]. Deviations from guideline recommendations may be justified in the context of medication shortages, allergy or adverse reactions to recommended antimicrobials, documented AMR or previous treatment failure, and clinical scenarios where guideline recommendations are absent or not applicable, such as in pediatric or immunocompromised populations [5,[8], [9], [10]]. Therefore, a more comprehensive conceptualization of appropriateness incorporates clinical context, patient-specific factors, and local prescribing realities, positioning appropriate prescribing as a structured clinical judgment encompassing indication, agent selection, dose, route, and duration, rather than a binary measure of guideline adherence.

The National Antimicrobial Prescribing Survey (NAPS) programme, developed in Australia, includes a model for appropriateness assessment that reflects this conceptualization. It has reported on the appropriateness of antimicrobial prescribing for more than a decade [11] and is a key requirement for hospital accreditation programmes [12]. It operates as a federally funded programme under the Australian Centre for Disease Control and contributes data to the AMU surveillance (Antimicrobial Use and Resistance in Australia surveillance programme [13]) and the AMR strategy [14]. Building on its success in Australia, the NAPS programme has been adapted and piloted in multiple international settings through a combination of collaborations including the National Centre for Antimicrobial Stewardship’s (NCAS) work with the WHO Collaborating Centre for Antimicrobial Resistance at the Doherty Institute, Melbourne, initiatives supported by the Fleming Fund Fellowship Programme [15], the Australian Department of Foreign Affairs and Trade, and direct partnerships with in-country organizations. Through these collaborations, pilots or implementations have occurred in Bhutan, Canada, Fiji, Malaysia, New Zealand, Pakistan, Papua New Guinea, Portugal, Timor-Leste, the United Kingdom, and Vietnam [[15], [16], [17], [18], [19]].

Expanding on this international experience, the Capturing Data on Antimicrobial Resistance Patterns and Trends in Use in Regions of Asia (CAPTURA) project [20], funded by the Fleming Fund [21], established a partnership between the International Vaccine Institute (IVI) (South Korea), the NCAS (University of Melbourne at the Doherty Institute, Australia), the Royal Melbourne Hospital Guidance Group (Royal Melbourne Hospital, Australia), and national stakeholders to implement or expand the NAPS programme in Bangladesh, Bhutan, and Nepal. Unlike earlier initiatives, which had already demonstrated the feasibility and adaptability of the NAPS programme in diverse international settings such as Malaysia, Canada, and Bhutan, this project sought to assess the feasibility and acceptability of implementation in new and markedly different health system contexts. Therefore, the CAPTURA implementation was designed as a pilot to evaluate how the NAPS programme could be locally sustained under varying resource, governance, and policy conditions. Demonstration of benefit and subsequent ministry endorsement would be prerequisites for broader institutionalization in each country.

Here we describe the implementation process of this scalable collaboration, its results, and practical lessons from these pilots, offering insights relevant to other countries seeking to strengthen AMU surveillance and AMS programmes. The programme was implemented within diverse health settings, each navigating challenges related to resourcing, infrastructure, and external disruptions linked to national events. Despite these challenges, the inherent adaptability of the programme, together with strong local commitment and adaptive implementation, enabled successful establishment and operation of AMU surveillance activities. These experiences demonstrate that, even in challenging and complex environments, meaningful impact can be achieved when a well-designed programme is supported by context-responsive planning and committed local leadership, improving AMU, enhancing patient safety, and contributing to global efforts to mitigate AMR.

## Methods

### Settings

Bangladesh, Bhutan, and Nepal were selected as candidate countries from the available Fleming Fund countries under the regional grant for the CAPTURA project, each representing different health system structures and levels of AMS maturity. Participating hospitals were identified as centers with relatively established clinical infrastructure and workforce capacity to support auditing. They were also considered potential centers for a hub and spoke model of AMS implementation, with the ability to lead new initiatives, demonstrate feasibility, and act as reference sites for scaling surveillance activities nationally, and will serve as exemplars from which other sites can adopt the approach. This will enable the generation of antimicrobial prescribing data to inform policy and stewardship priorities at a national level.

### Bangladesh

Bangladesh is a densely populated country with more than 170 million people, where rapid urbanization contrasts with a large rural population reliant on public health services. The publicly funded system includes 536 hospitals with 37,387 beds and 413 sub-district health complexes, most offering approximately 20 inpatient beds primarily for obstetric and emergency care [22]. Hospital capacity remains limited, with 0.9 beds per 1000 people in 2023 [23]. Despite expansion of secondary and tertiary facilities, shortages of specialists, diagnostics, and essential medicines persist. As of 2014, the most recent published data available at the time of writing, nearly 44% of physician posts and 96% of senior nursing positions were vacant, with deficits highest outside Dhaka [22]. Although incremental improvements have occurred in subsequent years, workforce shortages remain a significant structural challenge. Bangladesh has established a national framework for AMR containment, including a National Action Plan on AMR and dedicated institutional roles within national public health agencies. However, implementation of AMS activities at the hospital level remains uneven. Although earlier health sector reforms improved coordination with development partners, they also reinforced donor dependence [24]. These systemic barriers present major challenges for the implementation of AMS and surveillance activities.

### Bhutan

Bhutan is a small country with a dispersed population and government commitment to achieving universal access to healthcare. The Constitution guarantees free access to basic public health services in both modern and traditional medicine, with the state-funded system maintaining catastrophic health expenditure below 2% of household consumption [25]. The health system operates within a three-tier structure comprising 179 primary health centers, 53 sub-posts and 555 outreach clinics at the primary level, 49 district and general hospitals at the secondary level, and three national referral hospitals at the tertiary level [26]. Notably, More than 90% of the population lives within two hours of a health facility [26]. Hospital capacity remains limited, with 2.2 beds per 1000 people in 2023 [27]. Tertiary care is concentrated in a few urban centers, and some specialized treatment requires referral outside the country [26]. The Health Technology Assessment framework, first introduced in 2013, remains in the early stages of implementation and guides evidence-based prioritization of limited resources [26]. Despite these structural limitations, Bhutan has demonstrated commitment to AMR containment. The country endorsed a One Health–focused National Action Plan in 2017 and subsequently developed and implemented national antimicrobial prescribing guidelines in 2018. AMS programmes were established at the Jigme Dorji Wangchuck National Referral Hospital (JDWNRH) in 2016, followed by three additional hospitals in 2019—two regional referral hospitals and Phuentsholing General Hospital (PGH)—designated as sentinel surveillance sites for monitoring AMU under the Fleming Fund project [16]. Bhutan now has a national AMS and Infection Prevention and Control (IPC) center based at JDWNRH, with a dedicated team overseeing activities in the human health sector. However, fiscal limitations and workforce shortages remain significant barriers to the sustainability and expansion of these AMS and surveillance programmes.

### Nepal

Nepal has a population of approximately 30 million, with uneven distribution between densely populated urban areas and remote rural regions that limits equitable access to diagnostic and treatment services essential for AMR surveillance and control [28]. The health system operates under a federal structure that allocates responsibilities across federal, provincial, and local governments [29]. In 2021-2022, the public sector comprised 192 hospitals, 188 primary healthcare centers, and 3775 health posts, alongside 2155 non-public health institutions; however, access to functional microbiology laboratories remains limited and unevenly distributed [30]. Hospital capacity remains low, with 0.5 beds per 1000 people in 2023 [31]. Federalization has supported expansion of facilities, outreach services, and electronic reporting systems, but quality assurance and supervision remain limited [29]. The Nepal Health Facility Survey 2021 found that minimum quality-of-care standards were met in fewer than 1% of facilities, with 23.2% reporting any quality assurance activity and 4% maintaining client feedback mechanisms [29]. Deficiencies in coordination, procurement, and workforce distribution affect service delivery and undermine the sustainability of AMS programmes [32].

### The NAPS programme

The NAPS programme [16], developed by the Royal Melbourne Hospital and NCAS in Australia, is a clinician-centered, web-based auditing programme designed to measure AMU. It includes several audits tailored to different clinical settings. These include the Hospital NAPS [11], which evaluates inpatient antimicrobial prescribing through a point-prevalence audit, and the Surgical NAPS [33] that assesses antimicrobial prophylaxis prescribing before, during, and after surgery. All audits use standardized data fields to capture documentation quality, clinical indications, and antimicrobial choice.

Prescribing quality is evaluated using two clinical indicators: guideline concordance and overall prescribing appropriateness, a patient-centered assessment classified using standardized categories within each audit type. Additional process indicators, including documentation of indication and duration, are also captured.

These indicators form the basis of the NAPS programme’s reporting framework, enabling hospitals and health systems to monitor AMU and identify opportunities for quality improvement. The programme’s platform generates real-time, automated reports with benchmarking to support AMS activities and local policy development. Aggregated de-identified data contribute to national surveillance programmes and align with the WHO Global Action Plan on AMR [34], which identifies AMU surveillance as a core component of national AMR strategies. By providing standardized, comparable data, the programme facilitates evidence-based decision making, supports evaluation of AMS interventions, and strengthens national capacity to monitor progress toward AMR containment objectives.

The programme is delivered through a secure web-based platform that enables trained auditors to enter audit data directly through a structured online interface. The system uses role-based user permissions, with designated site or survey managers responsible for approving user access, managing audit activities, and overseeing data entry within their facility. The platform is cloud-hosted and accessible via standard web browsers, requiring only internet access and authorized user accounts. Participating facilities retain ownership of their data, with access to patient-level information restricted to local authorized users.

### Design and implementation

Activities were planned to be conducted over two phases in each country, spaced several months apart to allow time for interventions informed by baseline findings and to measure subsequent change. Sites were selected in consultation with national health authorities and stakeholders, with local implementers prioritized to support capacity building and sustainability beyond this initiative. Bangladesh and Nepal piloted the Hospital NAPS point-prevalence audit methodology, whereas Bhutan, where the Hospital NAPS had already been implemented nationally, piloted the Surgical NAPS to extend surveillance into the perioperative setting.

Four hospitals participated in Bangladesh; these were Shaheed Suhrawardy Medical College Hospital, Faridpur Medical College Hospital, Chattogram General Hospital, and Daudkandi Upazila Health Complex, representing tertiary-, secondary-, and district-level health facilities. Bhutan piloted the Surgical NAPS across four hospitals: JDWNRH, the largest referral hospital in the country, and three major regional hospitals, PGH, Central Regional Referral Hospital, and Eastern Regional Referral Hospital. In Nepal, the audits were implemented in two tertiary-care government hospitals: National Academy of Medical Sciences Bir Hospital, a large public facility in the capital, and Seti Provincial Hospital, a major provincial referral hospital.

A common implementation approach was applied across all countries. Ministries of Health, hospital leaders, and AMS and IPC committees were engaged from the outset to provide oversight and ensure national ownership. Selection of audit staff varied by country context. In Bangladesh, auditors were pragmatically selected from practicing physicians within each healthcare facility based on availability, ability to access patient medical records and prescribing documentation, and familiarity with clinical workflows. Preference was given to those actively involved in patient care and routinely engaged in prescribing or reviewing antimicrobial therapies. In Bhutan, the audit team was selected primarily from the AMS and IPC teams and included physicians, pharmacists, and nurses. Surgeons, who were not part of the AMS and IPC team, were also selected as auditors. They were engaged early during the pre-intervention phase to strengthen their understanding of AMS principles and to promote buy-in through direct involvement in the audit process. In Nepal, auditors were primarily nursing staff selected from hospital IPC units and AMS committees, prioritizing those with previous research experience, previous hospital-acquired infection surveillance and IPC activity involvement, and capacity to participate without disrupting routine duties.

All staff were trained in audit methodologies, including case selection, data entry, and application of standardized definitions; details of staff roles and training are presented in Table 1. Refresher workshops were delivered through scheduled and ad hoc sessions to reinforce consistency and address emerging questions during data collection. Data were collected using the standardized audit methodology for the respective audit type. For each prescription (Hospital NAPS) or course of prophylaxis (Surgical NAPS), appropriateness was categorized as ‘optimal’, ‘adequate’, ‘suboptimal’, ‘inadequate’, or ‘not assessable’, where ‘optimal’ and ‘adequate’ were considered ‘appropriate’. Appropriateness and guideline concordance assessments were undertaken collaboratively by the local multidisciplinary audit teams using standardized NAPS definitions and audit guidance. National or local guidelines were used where available as the primary reference for guideline concordance assessments. Where uncertainty arose during assessment, teams sought consultative guidance from the NCAS clinical support team to support consistent interpretation of complex cases. The NAPS methodology distinguishes guideline concordance from overall prescribing appropriateness, recognizing that prescriptions may remain clinically appropriate despite deviation from guidelines owing to patient-specific considerations, local antimicrobial availability, or limitations within existing guidelines, which may often be deemed outdated. The NCAS and IVI teams supported in-country implementation by providing training on audit methodology and AMS principles and clinical advice particularly for appropriateness assessments. They also developed data-cleaning guidance, conducted independent checks to ensure accuracy and completeness, and provided technical support as needed. The implementation adopted a train-the-trainer approach in principle, with the intention of progressively strengthening local expertise through repeated training and mentorship cycles. Over successive rounds, trained in-country teams and site audit managers were expected to take on greater responsibility for data entry oversight, feedback provision, and coordination between auditors and trainers as local capacity developed.

**Table 1:** Audit activity and training indicators across participating sites in Bangladesh, Bhutan, and Nepal.

Indicator	Bangladesh	Bhutan	Nepal
Number of facilities	4	4	2
Number of patients (Hospital NAPS) or procedures (Surgical NAPS) audited	446	236	853
Number of prescriptions audited	637	363	1308
Total number of staff trained	20	32	26
Type of staff trained	Doctors	Doctors, nurses, laboratory personnel, and pharmacists	Data analysts, doctors, and nurses
Training format	IVI in-person and NCAS online participation^a^ for workshops and audit support; NCAS ongoing remote technical support	IVI and NCAS in-person workshops and audit support; NCAS ongoing remote technical support	IVI and NCAS in-person workshops and audit support; NCAS ongoing remote technical support

Abbreviations: IVI, International Vaccine Institute; NAPS, National Antimicrobial Prescribing Survey; NCAS, National Centre for Antimicrobial Stewardship.

a NCAS staff participated remotely owing to Australian government travel advisories and security restrictions affecting travel to Bangladesh during the project period.

Training and implementation support were delivered through a combination of in-person workshops and remote technical support. In Bangladesh, initial workshops and on-site audit support were delivered in person by IVI trainers, whereas NCAS staff participated remotely owing to travel restrictions affecting travel from Australia to Bangladesh during the implementation period. In Bhutan and Nepal, IVI and NCAS staff jointly delivered in-person workshops and provided on-site support during audit implementation. Across all countries, additional technical support, clarification of audit procedures, and data validation guidance were provided remotely as required.

## Results

Within the three countries, 10 hospitals completed either Hospital or Surgical NAPS audits, in which a total of 1299 patients (Bangladesh 446, Nepal 853), 236 surgical procedures (Bhutan), and 2308 antimicrobial prescriptions were reviewed (Bangladesh 637, Bhutan 363, Nepal 1308) (Table 1). Seventy-eight hospital staff were trained (Bangladesh 20, Bhutan 32, Nepal 26), including doctors, nurses, laboratory personnel, pharmacists, and non-clinical support staff such as data analysts.

In Bangladesh, the first of two planned audit phases was completed between December 2024 and April 2025. The second phase was deferred owing to concurrent government reform processes and administrative transitions that temporarily affected institutional availability for external audit activities. Despite this, structured training and capacity-building sessions continued, ensuring that in-country auditors remained equipped to sustain antimicrobial prescribing audits beyond the formal project period. In Bhutan, audits were conducted within all participating sites between September 2024 and June 2025. All four sites completed the first audit phase. During the second phase, available funding and implementation capacity were prioritized to support continuation of audits at two key sites (JDWNRH and PGH), both of which successfully completed the second phase within the project period. In Nepal, audits were conducted in both sites simultaneously between March and September 2025, with both planned audit phases successfully completed during this period.

Key prescribing indicators generated through the audits are presented in Table 2. Overall, most prescriptions were prescribed empirically, and when broad-spectrum antimicrobials were used, these were more frequently assessed as inappropriate. Within the Hospital NAPS audits in Bangladesh and Nepal, surgical prophylaxis extending beyond 24 hours was common, with more than 50% of such prescriptions continuing past this duration. Incomplete clinical documentation was the most persistent barrier to prescription assessments, given that the indication for therapy or the rationale for antimicrobial choice was often not able to be ascertained. This hindered evaluation of appropriateness and guideline concordance, which was further complicated in settings lacking formal, written guidelines where clinicians instead relied on informal norms or verbal instructions from senior colleagues.

**Table 2:** Aggregated results of prescribing quality data for Bangladesh and Nepal.

Indicator	Bangladesh Hospital NAPS	Nepal Hospital NAPS
Prescriptions concordant with national or locally endorsed guidelines, n (%)	304 (47.7)	409 (31.3)
Appropriate prescriptions^a^, n (%)	482 (75.7)	659 (50.4)
Indication documented, n (%)	560 (87.9)	865 (66.1)
Stop or review date documented, n (%)	149 (23.4)	110 (8.4)
Surgical prophylaxis, ≥24 hours, n (%)	57 (44.5)	169 (63.5)

Abbreviation: NAPS, National Antimicrobial Prescribing Survey.

a Appropriate prescriptions were those assessed as ‘optimal’ or ‘adequate’.

At an operational level, the pilots demonstrated that audit methodologies must account for the realities of clinical documentation and hospital workflows. Logistical barriers included lack of formal antimicrobial approval mechanisms, paper-based or fragmented record systems, and intermittent network access, which compromised the timeliness of data collection. Documentation quality was the main determinant of audit completeness; incomplete or unclear records limited the ability to assess prescribing decisions. Some linguistic challenges were encountered, primarily arising from the use of an English-language audit tool by auditors from non-English-speaking backgrounds. These included uncertainty in understanding data entry parameters and terms such as ‘guideline concordance’ and ‘appropriateness’, particularly where up-to-date or locally relevant guidelines were absent, given that the latter is often used synonymously with guideline concordance elsewhere in the literature. Auditors suggested refinements to the data entry form, including clearer wording and an expanded indication list to better match local patient case mix. In some hospitals, auditors found it challenging to balance audit activities with clinical duties. Concerns were also raised about potential bias when auditors collected data in their own hospitals, which led to discussions about cross-hospital review mechanisms.

Hands-on refresher training demonstrated that auditors benefited from structured clarification on case classification and microbiology interpretation, and standardizing the distinction between guideline concordance and appropriateness emerged as a critical training outcome. Feedback from auditors informed refinements to trainer guidance and the development of decision aids. Participants also highlighted the digital data entry platform and automated dashboard outputs as particularly valuable; real-time results linked directly to clinical practice encouraged prescriber engagement and addressed limitations associated with paper-based audits.

## Discussion

The pilots of the NAPS programme in Bangladesh, Bhutan, and Nepal represent different stages of progress in national AMU surveillance and demonstrate what is required for antimicrobial prescribing audits to evolve from isolated exercises to sustained programmes. They provided a structured approach for collecting consistent data, building technical capacity, and strengthening institutional ownership of stewardship activities. In Bangladesh and Nepal, the audits generated the first national datasets describing prescribing practices, which also include the assessments of prescribing appropriateness as a key metric. In Bhutan, implementation of the Surgical NAPS extended an existing national programme into a new clinical domain, helping to identify perioperative stewardship priorities.

Governance and ownership were central to success. Where ministries of health and hospital AMS committees were actively engaged, audit findings translated into tangible actions such as revising protocols and establishing stewardship committees. These experiences emphasize that governance and national ownership are essential to sustain prescribing surveillance and translate data into improvement. Accreditation frameworks can provide a powerful governance mechanism to embed stewardship and surveillance activities into routine health service operations. In Australia, for example, the National Safety and Quality Health Service Standards require health services to maintain AMS programmes and undertake regular auditing of antimicrobial prescribing. This regulatory linkage between accreditation and AMS has driven the widespread adoption of surveillance systems such as the NAPS programme, demonstrating how policy-level levers can institutionalize stewardship practices and ensure accountability and sustainability.

Training played an important role in building confidence and consistency among auditors. Initial training established familiarity with the audit methodology, whereas repeated standardization and refresher sessions improved alignment in applying definitions and exercising clinical judgment, particularly in assessments of guideline concordance and appropriateness. Some auditors were also reluctant to classify prescriptions as non-concordant when they considered the treatment clinically appropriate, erroneously equating clinical appropriateness with guideline concordance, when these are in fact conceptually distinct measures. As described in the Methods, these are conceptually distinct: a prescription may be clinically appropriate despite deviating from guidelines owing to patient-specific considerations, local antimicrobial availability, or gaps within existing guidelines, and it is precisely this distinction that the appropriateness metric is designed to capture. Therefore, our findings reinforce the complementary value of recording both metrics and highlight that technical competence through ongoing training must be a key component of audit implementation.

Another important consideration is the availability of a workforce in each setting with the baseline skillset required to conduct the audit effectively. This includes clinical knowledge and experience, familiarity with reviewing clinical notes and medication charts, and an understanding of clinical workflows. These competencies are typically held by nurses, doctors, and pharmacists; however, their roles can differ depending on the country and context. For example, in some settings, the primary role of pharmacists is limited to dispensing rather than providing clinical services on the ward, which may have an impact on their ability to participate in stewardship activities. The ongoing shortage of staff to sustain routine clinical services already poses a significant challenge, and auditors reported that competing clinical duties often limit the time available for stewardship activities. This highlights the importance of management commitment and support to ensure appropriate scheduling, implementation of staffing standards [35], and supervisory oversight to maintain engagement and data quality. With sustained institutional commitment to AMS, these challenges can also create opportunities to expand the contribution of other professional groups [36] through targeted upskilling, thereby strengthening workforce capacity and embedding stewardship practices more broadly.

Linguistic factors were readily addressed through training. Although translation or local adaptation could further support clarity in future implementations [17], the pilots showed that effective audit delivery is achievable with appropriate training and oversight.

Implementation was also shaped by broader contextual factors, particularly in Bangladesh and Nepal, where administrative changes and periods of political unrest affected coordination and scheduling. Turnover in institutional leadership and shifting operational directives made it difficult to secure timely approvals and maintain continuity across project activities. These circumstances complicated the organization of trainings, meetings, and audit implementation, highlighting the need for adaptable planning, open and sustained communication among collaborators, and flexible timelines to preserve momentum under uncertain circumstances. In-country trainers also reported barriers linked to social and professional hierarchies, including deference to senior colleagues in the absence of formal guidelines, which required in-country trainers to navigate complex team dynamics and professional boundaries. Similar challenges have been noted in the literature, which highlights that AMS implementation in resource-limited, marginalized, or socio-culturally inequitable settings requires context-specific strategies that account for team hierarchies and interpersonal dynamics, including promotion of interdisciplinary collaboration [37,38].

Beyond these contextual factors, the operational and structural challenges identified in this study mirror barriers widely documented in the literature on AMS implementation in LMICs, including difficulties with paper-based or non-integrated record systems, limited microbiology capacity, staff attrition, incomplete prescription documentation, and low digitalization [37,39,40].

Although this project was not designed as a formal implementation science evaluation, the findings resonate with established frameworks for understanding stewardship implementation. Factors consistently identified in the implementation science literature [41,42] as enabling sustained programme adoption including local ownership, meaningful stakeholder engagement, adaptability to context, workforce capability, and integration within existing governance structures were each reflected in the experiences described across the three countries. Framing these findings within implementation science concepts such as sustainability, local adaptation, and iterative capacity building helps to situate this work within broader evidence base and may support transferability to other LMIC settings seeking to establish or strengthen AMU surveillance programmes.

Several limitations should be considered when interpreting these findings. A limited number of hospitals participated, purposively selected based on their capacity to support implementation and act as potential reference sites for future scale-up. Therefore, findings may not be representative of all healthcare settings within participating countries. Incomplete clinical documentation limited assessment of some prescriptions; however, this itself represented an important stewardship finding, highlighting documentation quality as an actionable target for improvement.

Sustainability remains a central challenge, given that competing priorities and limited resources can undermine the continuation of AMS initiatives once project funding ends. Integration of AMS activities, including AMU surveillance, into national systems with dedicated budget lines, clear institutional mandates, and leadership accountability is necessary to embed these activities within routine health service operations rather than treating them as time-limited projects. The technical capacity developed through this project provides a strong foundation, but cannot substitute for stable institutional and financial commitment. Accreditation frameworks, such as those linking AMS programme requirements to health service standards, offer one mechanism through which surveillance activities can be institutionalized and sustained beyond externally funded periods. Equally important is the quality of partnerships that ultimately enable local ownership. Progress depends on collaborations characterized by genuine commitment, shared goals, and mutual accountability. The partnership between NCAS, IVI, and in-country teams reflected these qualities, with stakeholders frequently contributing beyond formal expectations to ensure successful implementation. This dedication strengthened national capacity, fostered trust, and enhanced prospects for long-term sustainability. Future investments should prioritize these qualities, with particular emphasis on partnerships grounded in shared purpose. Funding mechanisms that recognize and reward these attributes will be critical to achieving sustained improvements in AMU and supporting national AMR strategies.

## CRediT authorship contribution statement

**Ron Cheah:** Conceptualization, Data curation, Formal analysis, Writing – original draft, Writing – review & editing, Project administration. **Karin Thursky:** Conceptualization, Formal analysis, Supervision, Writing – original draft, Funding acquisition, Writing – review & editing. **Jyoti Acharya:** Data curation, Formal analysis, Supervision, Writing – review & editing. **Andréia Regina Augusto dos Santos:** Formal analysis, Project administration, Writing – review & editing. **Sangita Shrestha:** Formal analysis, Writing – review & editing, Data curation. **Dorji Tshering:** Data curation, Formal analysis, Writing – review & editing. **Gitanjali Lamichaney:** Data curation, Formal analysis, Writing – review & editing. **Mohammad Julhas Sujan:** Data curation, Project administration, Writing – review & editing. **Hridika Talukder Barua:** Data curation, Project administration, Writing – review & editing. **Sonam Zangmo:** Data curation, Formal analysis, Supervision, Writing – review & editing. **Sanju Maharjan:** Data curation, Project administration, Writing – review & editing. **Samsad Rabbani Khan:** Data curation, Formal analysis, Writing – review & editing. **Siddhartha Dhungana:** Data curation, Formal analysis, Writing – review & editing. **Kumari Kamala Bist:** Data curation, Writing – review & editing. **Pem Chuki:** Data curation, Project administration, Writing – review & editing. **Khando Wangchuk:** Data curation, Writing – review & editing. **Abul Hasnat:** Data curation, Writing – review & editing. **Sooyoung Kwon:** Funding acquisition, Project administration, Writing – review & editing. **Zakir Hossain Habib:** Data curation, Writing – review & editing. **Santosh Paudel:** Writing – review & editing. **Manish Gautam:** Writing – review & editing. **Freddy Lopez:** Writing – review & editing. **Pradip Mishra:** Writing – review & editing. **Nimesh Poudyal:** Conceptualization, Funding acquisition, Supervision, Writing – review & editing. **Rodney James:** Conceptualization, Data curation, Formal analysis, Supervision, Writing – original draft, Writing – review & editing.

## Declaration of competing interest

The authors have no competing interests to declare.

## Appendix Supplementary materials

Image, application 1
