# Supplementary material for: Practical insights from implementing a national programme for monitoring appropriateness of antimicrobial prescribing in the South-East Asia Region: experience from the CAPTURA project in Bangladesh, Bhutan, and Nepal
Source: IJID Reg. 2026 Jul 10;20:100947. doi: 10.1016/j.ijregi.2026.100947 (PMC13475369; doi:10.1016/j.ijregi.2026.100947)
Supplement: Supplementary file 1 [file mmc1.docx]

SUPPLEMENTAL REFERENCE LIST

[1] Australian Medical Association. Antimicrobial resistance: the silent global pandemic. Canberra: Australian Medical Association; 2022. https://www.ama.com.au/antimicrobial-resistance (accessed March 6, 2026).
[2] McKenzie D, Rawlins M, Del Mar C. Antimicrobial stewardship: what's it all about? Aust Prescr 2013; 36: 116–20.
[3] Sulis G, Sayood S, Gandra S. Antimicrobial resistance in low- and middle-income countries: current status and future directions. Expert Rev Anti Infect Ther 2022; 20(2): 147–60.
[4] Barlam TF, Cosgrove SE, Abbo LM, MacDougall C, Schuetz AN, Septimus EJ, et al. Implementing an antibiotic stewardship program: guidelines by the Infectious Diseases Society of America and the Society for Healthcare Epidemiology of America. Clin Infect Dis. 2016; 62(10): e51–77.
[5] Lesho EP, Laguio-Vila M. The slow-motion catastrophe of antimicrobial resistance and practical interventions for all prescribers. Mayo Clin Proc 2019; 94: 1040–47.
[6] Markussen DL, Wathne JS, Ritz C, van Werkhoven CH, Serigstad S, Bjørneklett RO, et al. Determinants of non-adherence to antibiotic treatment guidelines in hospitalized adults with suspected community-acquired pneumonia: a prospective study. Antimicrob Resist Infect Control. 2024; 13(1): 140.
[7] Chuki P, Dorji T, James R, et al. Antibiotic use and quality indicators of antibiotic prescription in Bhutan: a point prevalence survey using the Australian National Antimicrobial Prescribing Survey tool. JAC Antimicrob Resist 2023; 5(4): dlad100.
[8] Mushtaq A, Cheah R, Luu S, Buising K. Evaluating the utilisation of the Quality Improvement National Antimicrobial Prescribing Survey (QI NAPS) at a public hospital in Pakistan. In: Australian Society for Antimicrobials Annual Scientific Meeting: Antimicrobials 2023; Brisbane, Australia; 2023. [abstract]
[9] Jamaluddin NAH, Periyasamy P, Lau CL, et al. Point prevalence survey of antimicrobial use in a Malaysian tertiary care university hospital. Antibiotics (Basel) 2021; 10(5): 531.
[10] Doshi J, Ngoc YP, Ma TT, et al. The effect of antimicrobial stewardship interventions upon antimicrobial consumption and appropriateness in Vietnamese district hospitals: a cluster randomised trial. Lancet Reg Health West Pac 2025; 60: 101620.
[11] Ng BY, Parsons T, O'Riordan B, Pill G, Dunsmure L, Jones N. P33 using the Australian National Antimicrobial Prescribing Survey (NAPS) portal to undertake an annual point prevalence survey (PPS). JAC Antimicrob Resist 2023; 5(Suppl 2): dlad066.037.
[12] Adhikari D. Healthcare and happiness in the Kingdom of Bhutan. Singapore Med J 2016; 57(3): 107.
[13] Dumka N, Gurung A, Hannah E, Goel S, Kotwal A. Understanding key factors for strengthening Nepal's healthcare needs: health systems perspectives. Journal of Global Health Reports 2024; 8: e2024010.
[14] Adhikari B, Ranabhat K, Khanal P, et al. Procurement process and shortages of essential medicines in public health facilities: a qualitative study from Nepal. PLOS Glob Public Health 2024; 4(5): e0003128.
[15] Twemanye V, Tumwine C, Mwanja H, et al. Implementation of the WHO point prevalence surveys on antibiotic use in a low-income country: experiences and lessons learned from Uganda. Wellcome Open Res 2026; 11: 9.
[16] Shamas N, Stokle E, Ashiru-Oredope D, Wesangula E. Challenges of implementing antimicrobial stewardship tools in low to middle income countries (LMICs). Infect Prev Pract 2023; 5: 100315.
[17] Kakumba JM, Kindenge JM, Kapepula PM, et al. Evaluation of antibiotic prescribing pattern using WHO access, watch and reserve classification in Kinshasa, Democratic Republic of Congo. Antibiotics 2023; 12: 1239.
[18] Zehra A, Ansari T, Shah SSAM, et al. Antibiotic stewardship benchmarking — using the WHO point prevalence survey of antimicrobial prescribing in a tertiary care public hospital, Karachi. PLoS One 2026; 21: e0342985.
